# Sub-clinical abnormalities detected by PET/MRI in household tuberculosis contacts

**DOI:** 10.1186/s12879-019-3705-0

**Published:** 2019-01-24

**Authors:** James S. Molton, Benjamin A. Thomas, Yan Pang, Lih Kin Khor, James Hallinan, Claire M. Naftalin, John J. Totman, David W. Townsend, Tow Keang Lim, Cynthia Bin Eng Chee, Yee Tang Wang, Nicholas I. Paton

**Affiliations:** 1 University Medicine Cluster, National University Health System, Singapore, Singapore; 2 Department of Medicine, Yong Loo Lin School of Medicine, National University of Singapore, Singapore, Singapore; 3 A*STAR-NUS Clinical Imaging Research Centre, Singapore, Singapore; 4 Department of Diagnostic Imaging, National University Health System, Singapore, Singapore; 5 Tuberculosis Control Unit, Tan Tock Seng Hospital, Singapore, Singapore

**Keywords:** Tuberculosis, PET-scan, MRI, Exposure

## Abstract

**Background:**

The understanding of early events following TB exposure is limited by traditional tests that rely on detection of an immune response to infection, which is delayed, or on imaging tests with low sensitivity for early disease. We investigated for evidence of lung abnormalities in heavily exposed TB contacts using PET/MRI.

**Methods:**

30 household contacts of 20 index patients underwent clinical assessment, IGRA testing, chest x-ray and PET/MRI scan using 18-F-FDG. MRI images were examined by a radiology/nuclear medicine dual-qualified physician using a standardised report form, while PET/MRI images were examined independently by another radiology/nuclear medicine dual-qualified physician using a similar form. Standardised uptake value (SUV) was quantified for each abnormal lesion.

**Results:**

IGRA was positive in 40%. PET/MRI scan was abnormal in 30%, predominantly FDG uptake in hilar or mediastinal lymph nodes and lung apices. We did not identify any relationship between PET/MRI findings and degree of exposure or IGRA status.

**Conclusion:**

PET-based imaging may provide important insights into the natural history following exposure to TB that may not be available from traditional tests of TB immune response or imaging. The clinical significance of the abnormalities is uncertain and merits further investigation in longitudinal studies.

## Background

It is increasingly recognised that patients infected with tuberculosis (TB) may have a spectrum of disease, from incipient disease to subclinical disease activity and eventually clinically-manifest active disease [1, 2]. Studies of the early manifestations of disease following TB exposure have been limited by the tools available. Traditional approaches for investigating patients for TB, which are limited to symptom screening, a chest x-ray (CXR) plus either a Tuberculin Skin Test (TST) or Interferon Gamma Release Assay (IGRA), provide poor discrimination of early events after exposure. Studies using Computed Tomography (CT) imaging have identified changes consistent with active TB in just under a third of TST or IGRA positive TB contacts [3–7].

PET-based imaging has shown particular promise for identifying clinically-significant abnormalities in asymptomatic patients with TB [8, 9]. In a study of HIV infected patients with latent TB, PET/CT also identified abnormalities consistent with subclinical TB in just under a third of cases [8]. Another small PET/CT study identified lymph node activity in four of five asymptomatic IGRA-positive TB contacts [9]. The aim of our study was to investigate a much larger group of contacts using PET magnetic resonance imaging (PET/MRI) to look for early abnormalities.

## Methods

The study recruited from July 2013 to April 2016. There were 2 recruitment sites, National University Hospital (NUH), and TB Control Unit (the national TB clinic which sees all the TB contacts in Singapore). At NUH, household contacts were approached through index pulmonary TB cases attending as inpatients. At TBCU, household contacts attending for routine screening as part of the national programme were approached about the study. Participants were eligible for inclusion if they were living in the same house as an index case (defined as an individual with smear positive pulmonary TB) for at least one month. Exclusion criteria were treatment for TB in the last year (treatment for LTBI was allowed), presence of metallic implants (contraindication for MRI) and poorly controlled diabetes mellitus (interference with uptake of the 18F-Fludeoxyglucose (FDG) used for PET scan).

Participants were asked about exposure-related factors: how long they had lived with the index patient; where they slept relative to the index patient; and how frequently they shared meals with them [10]. Participants were asked about any clinical symptoms suggestive of TB, smoking history and a physical examination was performed. Data relating to the index case (de-identified) was extracted from the national TB database (duration of cough, date of presentation, sputum smear grade, TB drug sensitivity and presence of cavitation on CXR. The time between exposure and imaging was estimated from the difference between the date the index patient first reported symptoms or began to live in the same household as the contact, whichever was later, and date of the of the PET/MRI scan.

The National Healthcare Group Domain Specific Review Board approved the study (2013/00116) including waiver of consent to collect de-identified information about the corresponding index patients from the national TB database. All participants gave written informed consent.

IGRA testing was performed using the QuantiFERON®-TB Gold In-Tube Test (QIAGEN, Hilden, Germany).

A standard posteroanterior CXR was performed. Where the routine hospital report identified abnormalities of consolidation, abnormal lymph nodes, pleural effusion or cavitation, x-rays were re-reviewed by a second (independent) radiologist. Where the originally reported findings were not confirmed on re-review, the CXR was regarded as normal.

### PET/MRI

PET/MRI was performed at the A*STAR-NUS Clinical Imaging Research Centre (CIRC) in Singapore using a Siemens Biograph mMR PET/MR scanner (Siemens Healthcare, Erlangen, Germany). Participants received an intravenous injection of 18F-FDG with a mean activity of 148·6 ± 54·7 MBq (mean ± s.d.). PET data was acquired for 15 min at 60 min post-injection (62·3 ± 12.6 min [mean ± s.d.]). The PET images were reconstructed using Ordinary-Poisson Ordered-Subset Expectation-Maximisation (OP-OSEM) with three iterations and 21 subsets. A Gaussian post-smoothing filter of 6 mm full-width at half maximum (FWHM) was applied. The matrix size was 172 × 172, with a voxel size of 4·17 × 4·17 mm and slice thickness of 2·03 mm.

The MRI data was acquired using 12-channel body coils. Dixon images were collected for the purpose of MR-based Attenuation Correction (MRAC). In addition, all subjects underwent T2-weighted half-Fourier acquisition single-shot turbo spin-echo (HASTE) with Prospective Acquisition CorrEction (PACE) and Diffusion Weighted Imaging (DWI).

### Image analysis

PET/MRI scans were read by two independent board certified dual trained radiology/nuclear medicine physicians (L.K.K. and J.H.) who did not have access to IGRA or other results. J.H. analysed only the MRI images, without having access to the PET images, while L.K.K independently analysed the combined PET/MRI images. Each reader recorded their findings on a standard data collection form that included the presence and location of consolidation, cavitation, pleural effusion, lymphadenopathy and nodules. For the purposes of the analysis of the MRI images alone, a lymph node of > 1 cm in short axis was considered enlarged and a nodule > 6 mm was considered significant. For the purposes of PET/MRI combined image analysis any lesion with standardized uptake value (SUV) > 0.95 was considered abnormal (based on a previous study using PET/CT that found that a cut-off value of 1.05 had 100% sensitivity and specificity for distinguishing active from inactive tuberculoma [11], and a study that showed SUV values using PET/MR are approximately 10% lower than PET/CT [12]). For each lesion the maximum SUV (SUVmax) was calculated.

The sample size of 30 was chosen based on pragmatic considerations of feasibility (recruitment and work of scans and analysis) and available funding, and was considered adequate to fulfil the descriptive objectives of the study. Statistical analysis was performed using SPSS version 21.

## Results

34 contacts were screened for the study and 30 were scanned (1 was excluded due to pregnancy and 3 withdrew consent before scanning). The median age was 42 (range 22 to 66) years; 43% were male; and the ethnic distribution was 47% Chinese, 33% Malay, 10% Indonesian, 7% Burmese and 3% Indian. Three contacts reported comorbidities (asthma, 1, gastritis, 1 and hypertension and hyperlipidaemia, 1). No contact reported a history of HIV or diabetes mellitus. Four were current smokers and one was an ex-smoker.

All contacts had slept in the same house as the index patient for a median of 20 years (range 2 months – 54 years). Of these, 13% slept in the same bed, 33% in separate beds in the same room and 53% in separate rooms; 60% ate together daily, 23% less than daily, and 17% never ate together.

The 20 index cases to which the contacts were linked were all sputum smear positive, of which 40% were grade ≥ 3+; all were culture positive; one had isoniazid resistance and the others had fully susceptible TB; 40% had cavitation on CXR.

Contacts were studied a median of 97 days (range 22–752 days) after estimated first TB exposure. Six contacts reported cough (duration one day to one year; CXR normal in all), three of which were productive of sputum (cultures negative for TB). No contacts had fever, night sweats, weight loss or haemoptysis.

The IGRA was positive in 12 (40%) contacts, negative in 15 (one of whom converted to positive on routine testing done 43 days later), indeterminate in one (tested negative on repeat testing 57 days later) and not analysed in two.

Four (13%) of the contacts had received isoniazid treatment for latent TB prior to imaging (median of 17 days, range 7–44 days). The CXR was abnormal in one contact (3%), showing minimal bilateral upper lobe scarring.

**Fig. 1:**
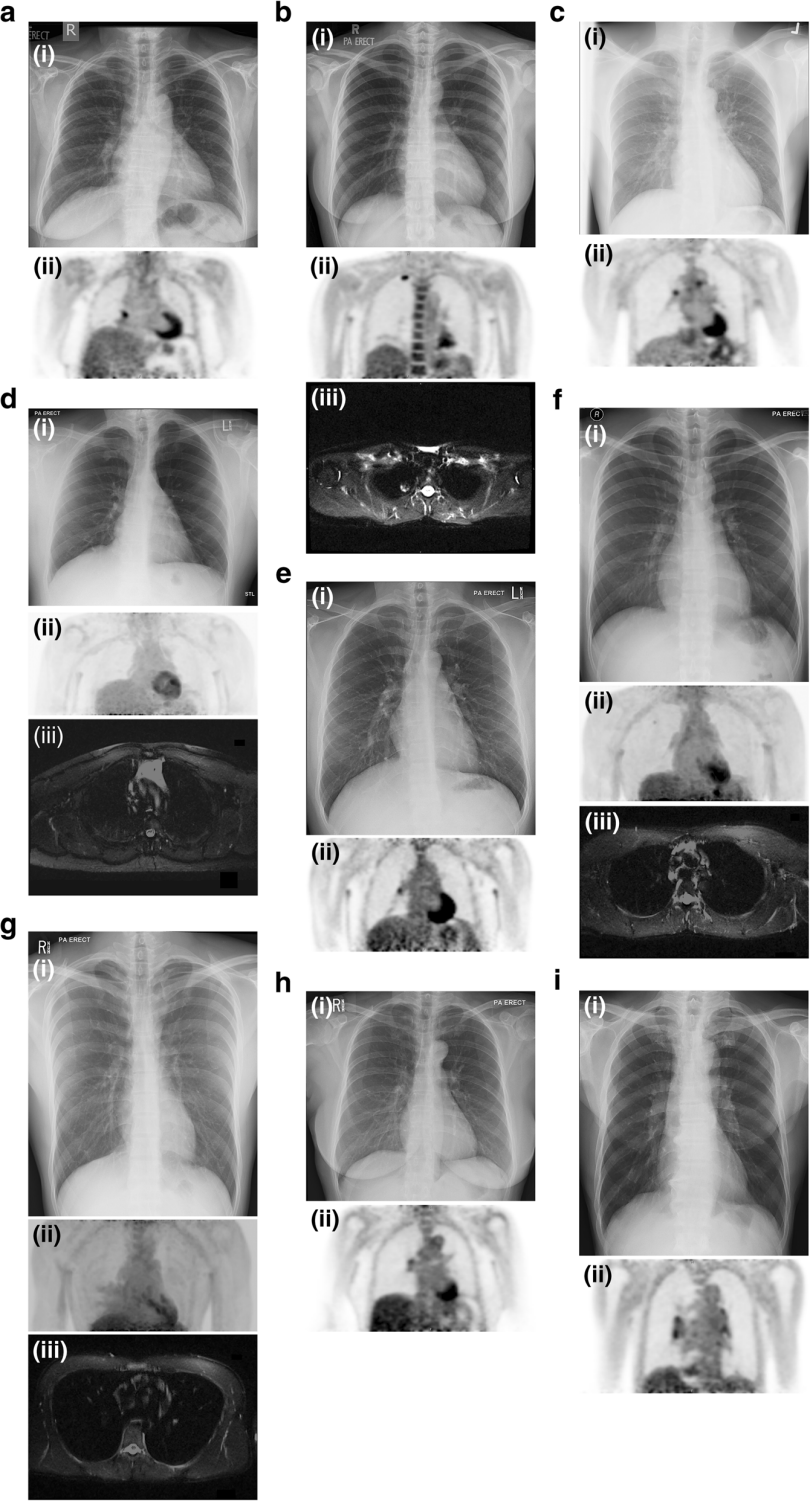
Abnormal imaging findings in TB contacts. **a**. Participant 2. 61y female, asymptomatic, (i) CXR: normal (ii) PET/MRI: FDG-avid right mediastinal lymph node (SUVmax 3.2); **b**. Participant 3. 35y female, asymptomatic, (i) CXR: normal (ii) PET/MRI: Spherical FDG-avid lesion in the apex of the right lung (SUVmax 3.9) (iii) Increased signal observed on MRI (HASTE); **c**. Participant 8. 54y male, asymptomatic, (i) CXR: normal (ii) PET/MRI: FDG-avid right hilar lymph node (SUVmax 2.4) and mediastinal lymph node (SUVmax 2.5); **d.** Participant 9. [914] 46y male, asymptomatic, (i) CXR: normal (ii) PET/MRI: Diffuse left upper lobe parenchymal uptake (SUVmax 2.2) (iii) No underlying MRI changes; **e**. Participant 12. 30y male, 1 week of mild non-productive cough, (i) CXR: normal (ii) PET/MRI: Right hilar lymph node (SUVmax 2.6); **f.** Participant 14. [922] 25y male, asymptomatic, (i) CXR: normal (ii) PET/MRI: RUL parenchymal uptake (1.3) (iii) Small underlying lesion identified on MRI images; **g.** Participant 16. [924] 22y male, 1 week of mild non-productive cough, (i) CXR: normal (ii) PET/MRI: Patchy right middle lobe parenchymal uptake (2.8), (iii) Bilateral underlying patchy changes identified on MRI. The area of right middle lobe uptake overlies an area of MRI abnormality; **h**. Participant 18. 46y female, asymptomatic, (i) CXR: normal (ii) PET/MRI: Right hilar lymph node (SUVmax 2.2); **i.** Participant 30. 66y male, asymptomatic, (i) CXR: normal, (ii) PET/MRI: several FDG-avid hilar lymph nodes (SUVmax = 2.8). (Note that all PET images are SUV images scaled between 0 (white) and 3 (black))

Analysis of the MRI images alone revealed only a 15 mm right upper lobe pulmonary nodule in one contact (Fig. 1 b (iii)) which was found to correspond to a PET-avid pulmonary nodule (SUVmax 3.9) at the same location on the independent analysis of the PET/MRI combined images. Two further contacts with normal PET/MRI scans (both never smokers) had nodules ≤6 mm detected on the review of the MRI alone, which were not considered significant.

The analysis of the combined PET/MRI images revealed abnormal metabolic activity in 9 (30%) participants (Fig. 1 a-f and Table 1). PET uptake was abnormal in all 9 (uptake in mediastinal lymph nodes, 3; hilar lymph nodes, 5; pulmonary nodule, 1; and other parenchymal lung uptake, 3). The SUVmax for these lesions varied from 1.3 to 3·9. Subtle MRI lesions were identified underlying the regions of FDG uptake in 2 out of the 3 contacts with parenchymal lung uptake (Fig. 1 f and g). In the other contact with parenchymal uptake, diffuse FDG uptake was seen in the anterior segment of the left upper lobe (SUVmax 2.2) but no underlying lung changes could be appreciated on MRI (Fig. 1 d).

**Table 1:** PET and MR findings in the 9 contacts with abnormal scans

Patient ID	Smoking history	Finding(s) on CXR	Finding(s) on analysis of MRI images alone	Findings on analysis of combined PET/MRI images (SUVmax)	Highest SUVmax	IGRA	Quantitative IGRA (IU/ml)
2	Never smoked	Nil	Nil	1 right hilar LN (3.2)	3.2	Positive	2.12
3	Never smoked	Nil	15 mm RUL nodule	1 RUL nodule (3.9) 1 mediastinal LN (3.1)	3.9	Positive	1.82
8	Current smoker	Nil	Nil	4 bilateral mediastinal nodes (2.1–2.5) 2 bilateral hilar nodes (1.6–2.4)	2.5	Positive	7.36
9	Never smoked	Nil	Nil	Diffuse LUL parenchymal uptake (2.2) No underlying MRI changes	2.2	Negative	0.01
12	Never smoked	Nil	Nil	1 right hilar LN (2.6)	2.6	Positive	0.58
14	Never smoked	Nil	Nil	RUL parenchymal uptake (1.3) Small lesion identified on MRI images underlying the FDG uptake	1.3	Negative	0.00
16	Ex-smoker	Nil	Nil	Patchy RML parenchymal uptake (2.8) Bilateral patchy changes identified on MRI. The area of RML uptake overlies an area of MRI abnormality	2.8	Negative	0.01
18	Never smoked	Nil	Nil	1 right hilar LN (2.2)	2.2	Negative	0.00
30	Never smoked	Nil	Nil	2 bilateral mediastinal LN (2.2–2.3) 8 bilateral hilar LN (2.6–2.8)	2.8	Negative	0.00

The one contact with the abnormal CXR was found to have bilateral pleural thickening and left apical scarring on MRI review, and an SUVmax of 1.9 on PET/MRI review. However this contact had a history of previously-treated TB which could explain these findings and this was not therefore considered an abnormality related to the recent TB exposure.

Of the 9 contacts who had abnormal PET/MRI scans, two had cough and four (44.4%) were IGRA positive. The 9 contacts with abnormal scans were linked to 8 index patients (median cough duration of 5 weeks (range 1–104 weeks); smear grade ≥ 3+ in four; cavitary disease in four). Two of the 9 contacts were on isoniazid treatment for latent TB at the time of the scan (for 13 and 44 days).

Of the 27 contacts with available data on index patient cough duration, 17 had exposure ≥90 days, 10 had exposure < 90 days. Scans were abnormal in 2/10 (20%) of those with short exposure and 6/17 (35%) of those with long exposure. The prevalence of scan abnormalities was 31% in the IGRA positive group and 33% in the IGRA negative group. There was no statistical association found between abnormal PET/MRI scan and history of cough (*p* = 1.00, 2-sided Fishers exact), intensity of exposure to index case (proximity of sleeping, (*p* = 0.405, 2-sided Fishers exact); frequency of eating together, (*p* = 0.253, 2-sided Fishers exact)), estimated duration of exposure, (*p* = 0.775, 2-tailed Mann-Whitney U), or smear status of index case (*p* = 0.110, 2-sided Fishers exact). There was no significant correlation between PET/MRI SUVmax values and initial quantitative IGRA values (Spearman r = 0.009; *p* = 0.979).

Across the whole cohort 11 contacts were ultimately treated for latent TB. A check of the national dispensing records revealed that no participants received treatment for active TB over a median of 2.2 years of follow up from date of scanning (range 1.2–3.9).

## Discussion

Using the new approach of PET/MRI, we identified increased metabolic activity in lymph nodes or lung parenchyma in approximately one third of heavily-exposed household TB contacts. Although there were few study eligibility criteria, healthy contacts who are motivated to enter a study and undergo a PET scan may differ from the general population of household contacts and this, together with the small sample size, means that the prevalence estimate may not be generalizable to the broader population of contacts. Nevertheless, the frequency of metabolic abnormalities appears much higher than the expected prevalence of active TB in this population (in a meta-analysis of 108 studies from high-income settings, the prevalence of active TB among contacts was only 1.4% [13]).

We did not scan a control population without household exposure to TB for ethical reasons and for funding and practical limitations. Most of the lesions had relatively low SUVmax values and interpretation of these depends on the background rate of lesions with low-level uptake in the normal population. A large study in Japan reported findings from 155,456 healthy people scanned with PET/CT [14]. Only 212 (0.14%) had some form of false positive inflammatory change in lung, and no lymph node false positives were reported. This supports our interpretation that the reported abnormalities in our study lie outside expectations for a healthy control population. In contrast, another study of 179 healthy controls scanned with PET/CT identified visually increased FDG uptake in hilar nodes in 28% of individuals [15]. However, none of these nodes had an SUVmax over 3, and even using this high threshold as the limit of the normal range would not negate our findings (two patients had higher SUVmax values; and the high threshold does not apply to parenchymal disease). One contact was identified as having diffuse FDG uptake within the lung parenchyma with no underlying structural abnormality on MRI, the significance of which is uncertain. This may be due to embolization of injected FDG, although this has previously been reported to produce a discreet focus of uptake rather than diffuse uptake [16]. While we cannot rule out all alternative explanations for the abnormal findings such as recent viral infection, TB is by far the most likely explanation given the circumstances (definite ongoing household exposure to a known case of TB, and a high IGRA positive rate (40%, compared to a rate of 12.7% in the local population [17]). Our findings are also plausible because studies using CT have also identified abnormalities in up to a third of TB contacts [3–7], and the nature and distribution of FDG uptake on PET/MRI is consistent with a study using PET/CT that reported lymph node activity in four of five TB contacts [9]. We did not find any statistical correlation between FDG abnormalities and degree or timing of exposure, which is surprising if the changes are due to TB exposure. This may be due to the small sample size.

The aetiology of the abnormal lesions that we observed is uncertain. They may represent an early stage of TB infection with active but low-level mycobacterial replication, sometimes referred to as “incipient disease” [2]. Another possibility may be that the FDG lymph node uptake represents transient immune activity following mycobacterial antigen presentation, which might potentially result in control or clearance of infection. The modest FDG activity (SUVmax 2–4) compared to active TB (typically 10–12) is consistent with either interpretation [18]. It is unknown whether presence of such early lesions might identify individuals at higher risk of subsequent progression to active disease. Asymptomatic HIV co-infected patients with abnormalities on PET/CT suggestive of subclinical TB had a higher risk of developing subsequent active clinical disease [8]. However, the lesions in that study were mainly parenchymal lesions, whereas we found mainly lymph node abnormalities. We did not systematically follow up the contacts, although offered a repeat scan in those with changes (three agreed to have repeat scans after an interval of 6 months (range 182–193 days) but showed no evidence of progression). We also searched the national treatment database for longer term outcomes and found that none had relapsed. However the median duration of follow up (2 years) was relatively short and these findings might confer a higher risk of relapse in the longer term. Although HIV was not tested as part of the study, no contact had known HIV and the background prevalence of HIV in the local population is only 0.001% [19]. In a population with a low overall risk of reactivation, and a small sample size, the absence of any cases of observed progression does not negate the possibility of increased risk of re-activation in those with PET/MRI abnormalities (larger studies would be needed with more practical biomarkers to determine this, see below).

The high prevalence of abnormalities we observed are even more remarkable given that we did not limit recruitment to contacts who were IGRA positive; we recruited independent of IGRA status because we did not want to assume a priori that PET/MRI would be normal in those who did not mount an IGRA response following TB exposure. Indeed there was no correlation between IGRA and scan abnormalities. The IGRA test is based on the T-cell response to infection and the observed PET activity also most likely reflects the immune response as uptake by metabolically-active bacilli is likely small given the low bacillary burden in this early disease stage. The lack of concordance between the two investigations may simply be due to the small sample size, but might also reflect the difference between the standardised antigenic stimulation with IGRA [20], and the natural immune response which is likely to evolve between exposure and scan. Although the scans were performed promptly after the identification of a contact, the time for the index case to present introduced an unavoidable delay in time between first exposure and scan, during which resolution may have occurred. MRI has higher sensitivity than CT for detecting nodal involvement in patients with active pulmonary TB [21]. Previous studies investigating PET in TB have all used PET/CT. As lymph nodes appear to be the commonest site of subclinical pulmonary activity in TB contacts, PET/MRI may possibly be a preferred imaging modality over PET/CT in research studies in TB contacts. PET/MRI has the additional advantage of lower radiation dose than PET/CT, which may be important for research applications in healthy contacts.

## Conclusions

Given the cost, radiation dose and practical challenges involved in implementing at scale in large numbers of contacts, it is unlikely that PET-based imaging will find a clinical role in the screening of TB contacts. However, it may have considerable value in research studies for defining underlying differences in disease status that are missed by the traditional clinical screening methods. The ability to characterise clinical status in more detail may provide a foundation for future studies to identify a simpler and cheaper biomarker that may reflect these subclinical changes seen on PET/MRI. However prior to this larger PET imaging studies would be necessary to confirm if the population with subclinical FDG uptake indeed represents a population at higher risk of future reactivation. Such biomarkers could then be applied on a much larger scale for testing TB contacts and following outcomes, to determine whether these changes are predictive of relapse.

## Abbreviations

CT: Computed Tomography
CXR: Chest X-ray
DWI: Diffusion weighted imaging
FDG: 18F-Fludeoxyglucose
FWHM: Full-width at half maximum
HASTE: Half-fourier acquisition single-shot turbo spin-echo
HIV: Human immunodeficiency virus
IGRA: Interferon gamma release assay
MRAC: Magnetic resonance based attenuation correction
MRI: Magnetic resonance imaging
OP-OSEM: Ordinary-poisson ordered-subset expectation-maximisation
PACE: Prospective acquisition correction
PET: Positron emission tomography
PET/CT: Positron emission tomography computed tomography
PET/MRI: Positron emission tomography magnetic resonance imaging
SUV: Standardized uptake value
SUVmax: Maximum SUV
TB: Tuberculosis
TST: Tuberculin skin test
